# A month for celebration

**DOI:** 10.1177/17504589231165366

**Published:** 2023-05-05

**Authors:** Victoria Cadman

This month, many of our colleagues will be celebrating their respective professions. Nurses have ‘International Nurses Day’ on the 12th, closely followed by Operating Department Practitioners’ (ODPs) ‘National ODP Day’ on the 14th. These are important days in each profession’s calendar as they allow opportunity for us to recognise our achievements and publicly acknowledge the valuable work that we do caring for society.

For ODPs in particular, our national day is also a perfect opportunity for us to raise public awareness of the profession. While this is also important for nurses in terms of awareness of scope of practice, for ODPs who are not as far along the ‘professional journey’ as nurses, it is perhaps more so. ODPs have come a long way in the last 20 years since becoming an Health and Care Professions Council (HCPC) regulated profession in 2004, gaining recognition as an allied health profession in 2017 and transitioning from a diploma to a degree award qualification. The National ODP Day that was initiated in 2018 finally gave us a date to honour our profession. For a role that is predominately behind closed theatre doors, National ODP Day is a chance for ODPs to come from behind those doors and move to more visible areas such as hospital entrances, schools and colleges, university spaces and shopping centres to promote the profession and inspire future generations of our workforce. In a year where professions are still dealing with the impact of the COVID-19 pandemic, coupled with negative perceptions portrayed in the media, this may prove more challenging than most, yet equally more important as a result. Preparations for events held around our celebratory days will therefore also give us valuable time to reflect and remind ourselves of the many positives of our work, what inspired our own journeys into the role, and the evolving opportunities and career progression we now have thanks to our predecessors.

So, whatever you may be doing on International Nurses Day or National ODP Day, be it a celebration in the coffee room, display stand and bake sale in the hospital entrance, or activities explaining the role to your local school or youth group, have fun celebrating. From all of us at the Association for Perioperative Practice and Journal for Perioperative Practice, thank you for all that you do.


Victoria Cadman Associate Editor, Journal of Perioperative Practice V.Cadman@shu.ac.uk


